# Referral patterns, delays, and equity in access to advanced paediatric emergency care in Vietnam

**DOI:** 10.1186/s12939-017-0703-y

**Published:** 2017-12-15

**Authors:** Emily Treleaven, Toan Ngoc Pham, Duy Ngoc Le, Trevor N. Brooks, Hai Thanh Le, J. Colin Partridge

**Affiliations:** 1 Department of Social and Behavioral Sciences, University of California, 3333 California St., Ste. 455, San Francisco, CA 94143-0612 USA; 2 Department of Emergency Medicine, Vietnam National Children’s Hospital, Hà Nội, Vietnam; 3 University of California, San Francisco School of Medicine, 513 Parnassus Ave, San Francisco, CA 94143 USA; 4 Department of Pediatrics, University of California, Mission Hall 5th Floor, 550 16th Street, San Francisco, CA 94143 USA

## Abstract

**Background:**

Quality emergency care is a critical component of a well-functioning health system. However, severely ill children often face barriers to timely, appropriate care in less-developed health systems. Such barriers disproportionately affect poorer children, and may be particularly acute when children seek advanced emergency care. We examine predictors of increased acuity and patient outcomes at a tertiary paediatric emergency department to identify barriers to advanced emergency care among children.

**Methods:**

We analysed a sample of 557 children admitted to a paediatric referral hospital in Hanoi, Vietnam. We examined associations between socio-demographic and facility characteristics, referrals and transfers, and patient outcomes. We used generalized ordered logistic regression to examine predictors of increased acuity on arrival.

**Results:**

Most children accessing advanced emergency care were under two years of age (68.4%). Pneumonia was the most prevalent diagnosis (23.7%). Children referred from lower-level facilities experienced higher acuity on arrival (*p* = .000), were more likely to be admitted to an ICU (*p* = .000), and were more likely to die during hospitalization (*p* = .009). The poorest children [OR = 4.98, (1.82–13.61)], and children entering care at provincial hospitals [OR = 3.66, (2.39–5.63)] and other lower-level facilities [OR = 3.24, (1.78–5.88)] had significantly higher odds of increased acuity on arrival.

**Conclusions:**

The poorest children, who were more likely to enter care at lower-level facilities, were especially disadvantaged. While delays in entry to care were not predictive of acuity, children referred to tertiary care from lower-level facilities experienced worse outcomes. Improvements in triage, stabilization, and referral linkages at all levels should reduce within-system delays, increasing timely access to advanced emergency care for all children.

## Background

A quality emergency care system, including referrals and transfers to appropriate care, is a critical component of a well-functioning health system. However, cumulative delays in care seeking, diagnosis, and referral for tertiary care contribute to infant and child morbidity and mortality, even where well-developed emergency medical systems exist [1–3]. In places with less developed medical systems, illnesses such as pneumonia may become life-threatening if children do not access quality, appropriate care in a timely manner. In these settings, many children face social and structural barriers to care, and experience additional delays where clinical capacities are poorly developed [4–7]. These barriers are particularly acute among the poorest children [8, 9]. Children with higher acuity on arrival require more intensive care and medical resources, and face increasing risk of mortality. Emergency care is often one of the weakest components in less developed health systems [10]. However, equity of access to and utilization of advanced paediatric emergency care in these settings is not well understood, requiring further study to identify areas for improvement.

In most countries, tertiary emergency care is typically only available at select hospitals in major urban centres. In many lower- and middle-income countries, primary and secondary facilities may lack sufficient adequately skilled providers, equipment, or technology to provide intensive care for children [1, 5, 11]. Triage systems are often limited at mid-level facilities, where providers may not recognize severe illness in a timely manner [12–15]. Paediatric emergency and critical care evidence-based guidelines, such as the Emergency Triage Assessment and Treatment (ETAT) guidelines exist, yet are often poorly implemented at facilities at all levels [5, 16, 17]. Poor implementation of such guidelines may lead to lower quality, sub-standard care.

In addition to issues of quality and availability of care, delays further complicate children’s ability to reach appropriate emergency care. Parents and caregivers may defer care seeking until disease is advanced, or may not recognize when symptoms are severe, especially in the case of pneumonia [18, 19]. Structural barriers, such as cost and transportation, and social barriers, such as low health literacy and gender norms that disadvantage women and girls, also contribute to delays in entry to care [6, 7, 20]. Schemes to improve health equity, such as the removal of user fees, may mitigate some of these barriers to access, specifically cost [21, 22].

When children first enter care, they may face further delays to treatment if they require referral. Emergently ill children often first present to outlying, lower-level facilities that lack paediatric expertise to quickly diagnose, triage, stabilize or appropriately treat high acuity paediatric patients [2]. For many of the same reasons that children face challenges in accessing primary and secondary facilities, such as cost and distance, referrals to tertiary facilities are also difficult [23]. A lack of transportation between facilities may present a key barrier, especially for families required to provide their own transport to referral facilities [24, 25]. When ambulance transfer is provided, releasing a doctor or nurse to accompany a critically ill child poses a challenge for facilities with limited staff [1]. Even in well-defined referral systems, compliance with referrals may be low if families cannot bear the cost of further treatment [26]. Inefficiencies in the health system and issues of communication between providers and parents also contribute to delays in care [12]. Such system-related barriers disproportionately affect the poorest patients. These patients face more difficulties in navigating care and referrals, and communicating with health providers, and are more likely to experience delays after entering care [12]. They may be more likely to experience increased acuity on arrival at referral facilities as a result of these system-related delays, potentially contributing towards higher morbidity and mortality among disadvantaged patients.

An examination of how severely ill children access highest-level emergency care in less-developed health systems, including equity of access to needed care, patient outcomes, characteristics of referrals and transfers, and drivers of higher acuity on arrival, can inform an understanding of how these systems function and how they might be improved. In this study, we aim to analyse how access to advanced paediatric emergency care contributes to child health disparities in a developing health system in order to identify areas for intervention. Given that poorer children are significantly more likely to enter care at primary or secondary facilities [27], they may be especially disadvantaged in accessing quality, appropriate emergency care compared to richer and urban children. Thus, identifying characteristics of care seeking, referrals, transfers, and other dimensions of healthcare that contribute to severity of illness on arrival is especially salient to understanding and addressing children’s disparities in access to emergency care.

We study these issues in Vietnam, a lower-middle income country with a pluralistic, increasingly complex health system. Vietnam has a hierarchical public health system that offers advanced paediatric critical and emergency care in major cities. The public-sector healthcare system is comprised of community health centers and polyclinics, and district, provincial, and national-level government hospitals. Referrals typically occur sequentially from community facilities to district-level facilities, provincial-level hospitals, and finally to national-level hospitals for increasingly higher-level care. Provincial and national-level hospitals may be accessed directly if parents are willing to pay additional costs. Community-level facilities provide primary or secondary-level care, while district, provincial, and national hospitals offer tertiary care. However, compared to national hospitals, district and provincial hospitals provide a lower quality of care. In Vietnam, a thriving private sector provides care at all levels. Private-sector clinics are found throughout the country, including in rural areas, and the number of private hospitals is growing in metropolitan areas [28]. However, the private sector is poorly regulated, and quality of care in these facilities is generally poorer [28, 29]. Currently, Vietnam has a limited number of evidence-based recommendations for paediatric emergency care, and even more limited guidelines for emergency transport.

Since 2005, a government scheme has exempted children under six years of age from user fees at public facilities [30]. Another government scheme, implemented in 2002, seeks to reduce the financial burden of care for the poor and ethnic minorities [31]. In general, these subsidies have increased utilization of public sector care among the poor, including at secondary and tertiary facilities [31, 32]. However, how these increases in access translate to advanced paediatric emergency care is unknown.

In this study, we identify socio-demographic and medical risk factors, facility characteristics, and health system factors associated with higher acuity on arrival to tertiary emergency medical care among children. We pay special attention to pneumonia, an important cause of child mortality globally and a disease that disproportionately affects poorer children [33, 34]. We describe referral patterns to highest-level care, and patient outcomes at a tertiary facility.

## Methods

### Study setting

This study was conducted at Vietnam National Children’s Hospital (VNCH), a national public-sector paediatric referral hospital in Hanoi. VNCH accepts neonates, infants, and children on a self-referral basis or referred from public and private providers for advanced paediatric care, including emergency care. A majority of injury and trauma cases are referred elsewhere.

### Data sources and collection

We conducted a prospective cohort study of 585 paediatric patients under age 18. The sample included patients admitted to the hospital after presenting to the Emergency Department (ED) from August to December 2013. During the study period, every fourth patient who met study inclusion was approached for enrolment. Eligible patients admitted through the ED were identified within 12 h of admission and approached for enrolment within 48 h of admission.

On arrival at the ED, a trained nurse assigned each patient an acuity score using a validated acuity assessment tool, the Emergency Severity Index Version 4 [35]. Patients with moderate or high acuity scores (ESI 1 [imminent death], 2 [immediate need for intervention], or 3 [need for multiple interventions]) who were admitted to an in-patient ward or intensive care unit (ICU) were eligible. For ethical reasons, we excluded patients who died in the ED or who were imminently dying on ED admission (*N* = 17).

Interviewers were trained in informed consent, study processes and instruments, and techniques for interviewing when emergency situations might engender emotional distress. Accompanying parents or caregivers provided written informed consent (76.7% mother, 15.9% father, 4.8% grandparent, 2.6% other relative). Among those approached, 98.4% agreed to participate. Participants self-administered a 42-item questionnaire (94.5%) or were administered the questionnaire by an interviewer (5.4%). The survey included information on socio-demographic characteristics, care seeking for the child, and transport. Hospital chart review was conducted to collect admission and outcome data, including diagnosis (ICD-10 code). Participants were followed to death (*N* = 2), withdrawal of support (*N* = 13), or hospital discharge (*N* = 542).

We excluded 28 children from analysis for whom information on diagnosis, outcomes, or other relevant characteristics was incomplete. The final analytical sample included 557 children.

We used a dichotomous measure of whether the child was referred from a provincial hospital versus other facility, versus entering care directly at VNCH, based on medical records. Delay of care seeking was calculated as the difference in parents’ report of the time of recognition of illness and when care was first sought outside the home, measured in days. Children’s age was dichotomized as five and under versus six years of age or older to capture whether the child was eligible for the user fee exemption. We used a dichotomous measure of child’s sex (male versus female). Children’s diagnosis is categorized as whether the child had pneumonia, diarrhoea, or another illness (ICD-10 code). Parents self-reported urban versus rural residence, and maternal educational attainment, categorized as any secondary or any post-secondary education versus primary schooling. Household wealth quintiles were constructed from the full dataset using principal components analysis of standard household asset indicators, an approach used in large household surveys in Vietnam, such as the Multiple Indicator Cluster Survey [36, 37]. In regression analyses, dummy variables for each wealth quintile were entered with the highest quintile as a reference category, with an additional dummy for children whose household wealth is missing (*N* = 90).

### Data analysis

In bivariate analyses, we examined associations between acuity on arrival, referrals, transfers, and outcomes. We then examined these associations among children diagnosed with pneumonia or diarrhoea versus all other conditions. We tested for bivariate associations using chi-square and t-tests depending on the outcome of interest. For non-parametric outcomes, we used Kruskal-Wallis tests.

In regression analysis, the primary outcome variable was acuity score at time of admission to the ED as measured using ESI. We used a generalized ordered logistic regression to examine predictors of increased acuity on arrival, based on a priori hypotheses of social, physical, and health system determinants. Covariates in the regression analysis are described above, apart from diagnosis. In regression analyses, we dichotomize type of illness as pneumonia versus other as there is not sufficient variation in acuity among children with diarrhoea; therefore, we group them with the other illness category. This analysis was performed using *gologit2*, a generalized ordered logit model with a partial proportional odds assumption [38] in Stata Version 14.1. We constrain all independent variables except pneumonia diagnosis to meet the parallel lines assumption across the levels of the dependent variable, ESI score. This modelling strategy accounts for the non-proportional difference in acuity scores from moderate to highest acuity, and is more parsimonious than a multinomial logistic regression model. We tested interactions between rural residence, age, sex, and wealth status. An interaction term for whether the child was exempt from user fees and from a rural area was significant and included in the final model. We examined alternative categorizations of age, maternal education, and delays to care to assess sensitivity. The resulting estimates did not change our findings substantively.

### Ethical considerations

The child’s parent or an accompanying caregiver provided written informed consent for all study participants. Children over age seven provided written assent. Approval for the study was obtained from the Institutional Review Boards of the University of California, San Francisco, and the Vietnam National Children's Hospital.

## Results

The sample included 557 paediatric patients presenting to the ED with ESI scores of 1 to 3 who were admitted to hospital wards or intensive care units (Table 1). Of 557 patients, 59 (10.6%) had an ESI score of 1 (highest acuity) on arrival at the ED, 164 (29.4%) had an ESI score of 2 (high acuity), and 334 (59.9%) had an ESI score of 3 (moderate acuity). The sample had a high proportion of boys (67.7%), however, the ratio of boys to girls in our sample is consistent with overall hospital admissions. A majority of children were less than two years of age. About half resided in a rural area. Almost all children were Kinh, or ethnic Vietnamese (95.9%). Maternal educational attainment was high, with 23.9% of mothers attending secondary school, and 40.0% of mothers attending college or technical or vocational school. Pneumonia was the most commonly diagnosed illness (23.7%). Almost 10% of children in the sample were diagnosed with diarrhoea. Among children diagnosed with other illnesses (66.8%), the most prevalent diagnoses included acute appendicitis (*N* = 25), intussusception (*N* = 22), and acute bronchiolitis (N = 22).

**Table 1:** Sample characteristics (*N* = 557)

	*N* (%)
Child sex	
Male	377 (67.7%)
Female	180 (32.3%)
Mean age in years (SD)	1.8 (2.7)
Age	
Newborn (<28 days)	6 (1.1%)
1–11 months	245 (44.0%)
12–23 months	130 (23.3%)
2–5 years	111 (19.9%)
6–9 years	48 (8.6%)
10–17 years	17 (3.1%)
Diagnosis	
Pneumonia	132 (23.7%)
Diarrhoea	53 (9.5%)
Other illnesses	372 (66.8%)
Mother’s median age in years (SD)	29.3 (5.5)
Father’s median age in years (SD)	32.8 (6.4)
Mother’s highest educational attainment (*N* = 553)	
Primary	70 (12.7%)
Lower secondary	130 (23.5%)
Upper secondary	132 (23.9%)
Technical/vocational	47 (8.5%)
College/university	174 (31.5%)
Father’s highest educational attainment (*N* = 555)	
Primary	67 (12.2%)
Lower secondary	138 (25.2%)
Upper secondary	149 (27.2%)
Technical/vocational	20 (3.7%)
College/university	174 (31.8%)
Mean household size (SD)	4.6 (1.6)
Resides in a rural area	287 (51.5%)
Kinh (Vietnamese ethnicity)	534 (95.9%)
Wealth quintile	
Poorest	96 (17.3%)
Poor	91 (16.3%)
Middle	96 (17.2%)
Rich	88 (15.8%)
Richest	96 (17.2%)
Missing	90 (16.2%)

### Bivariate analyses

Several socio-demographic characteristics were significantly associated with higher acuity. Poorer children were significantly more likely to experience high or highest acuity on arrival (*p* = .017). Younger children had significantly higher acuity on arrival (*p* = .000, data not shown), as did children from rural areas (*p* = .003).

Examining children’s care seeking and outcomes by acuity (Table 2), sicker children were significantly more likely to delay care seeking outside the home (*p* = .039). Less than one-fifth of all children sought care outside the home within 24 h (15.4%), and 14.2% waited more than one week. Children with moderate acuity (ESI 3) had the lowest mean delay to care (3.6 days, SD = 4.7), while those with high acuity (ESI 2) had the longest mean delay (4.7 days, SD = 5.8). Overall, 15 children died or had support withdrawn. Most children were discharged home (96.9%), while two were discharged to a lower-level facility.

**Table 2:** Care-seeking behaviours and outcomes by severity of illness on admission

	Total (N = 557)	ESI 1 (*N* = 59)	ESI 2 (*N* = 164)	ESI 3 (*N* = 334)	*p*-value
Mean time from recognition of illness to seeking care outside the home (days) (SD)	4.0 days (5.2)	4.2 days (5.8)	4.7 days (5.8)	3.6 days (4.7)	*p* = .039
Days delayed care seeking					*p* = .014
Less than 24 h	86 (15.4%)	11 (18.6%)	16 (9.8%)	59 (17.7%)	
24–47 h	146 (26.2%)	15 (25.4%)	46 (28.1%)	85 (25.5%)	
48–71 h	65 (11.7%)	1 (1.7%)	15 (9.2%)	49 (14.7%)	
3–7 days	181 (32.5%)	23 (39.0%)	55 (34.2%)	102 (30.5%)	
More than one week	79 (14.2%)	9 (15.3%)	31 (18.9%)	39 (11.7%)	
Admitted to an ICU	48 (8.8%)	16 (27.1%)	23 (14.0%)	10 (3.0%)	*p* = .000
Died in hospital	15 (2.7%)	5 (8.5%)	5 (3.1%)	5 (1.5%)	*p* = .009
Referred to VNCH from lower-level facility	211 (38.0%)	37 (62.7%)	88 (53.7%)	86 (25.8%)	*p* = .000
Referring facility level (*N* = 211)					*p* = .955
Provincial	146 (69.2%)	27 (73.0%)	62 (70.5%)	57 (66.3%)	
District	29 (13.7%)	6 (16.2%)	11 (12.5%)	12 (14.0%)	
Commune	0 (0%)	0 (0%)	0 (0.0%)	0 (0%)	
Private	6 (2.8%)	1 (2.7%)	3 (3.4%)	2 (2.3%)	
Other	13 (6.2%)	1 (2.7%)	6 (6.8%)	6 (7.0%)	
Don’t know	17 (8.1%)	2 (5.4%)	6 (6.8%)	9 (10.5%)	
Transferred in ambulance (N = 211)	122 (57.8%)	28 (75.7%)	65 (73.9%)	29 (33.7%)	*p* = .000
Staff accompanied in ambulance (N = 211)	90 (49.6%)	20 (54.5%)	47 (53.4%)	23 (26.7%)	*p* = .000
Doctor accompanied	16 (7.6%)	3 (8.1%)	8 (9.1%)	5 (5.8%)	
Nurse accompanied	74 (35.1%)	17 (46.0%)	39 (44.3%)	18 (20.9%)	
None	22 (10.4%)	6 (16.2%)	13 (14.8%)	3 (3.5%)	
Don’t know/missing	6 (2.8%)	11 (29.7%)	28 (31.8%)	60 (69.9%)	

Less than two-fifths of children in the sample were referred from a lower-level facility (38.0%, Table 2). Children referred from a lower-level facility were significantly more likely to have higher acuity than children who entered the ED directly (*p* = .000). Among the 211 children who entered care at a lower-level facility, the majority were referred from a provincial hospital (69.2%), followed by district-level facilities (13.7%). Few children were referred from private facilities (2.8%). Among all referred children, 57.8% were transferred in an ambulance, with highest and high acuity children significantly more likely to receive ambulance transport than children with moderate acuity (*p* = .000). Few children (7.6%) were accompanied by a doctor in ambulance transfers. Sicker children were significantly more likely to be accompanied by a doctor or nurse in an ambulance (*p* = .000). Among those who were referred, the poorest children were significantly more likely to be transferred by ambulance than the richest (*p* = .02), and ethnic minorities were more likely to be transferred by ambulance than ethnic Vietnamese children (*p* = .08, data not shown).

Children referred from another facility experienced worse outcomes than children who entered care at the ED. In addition to higher acuity, these children had a significantly greater likelihood of being admitted to an ICU (*p* = .000, data not shown). Compared to children who entered care at the ED, children who were referred were significantly more likely to die in care or have support withdrawn than to be discharged (*p* = .001).

Children with pneumonia were significantly more likely to delay seeking care outside the home than children with diarrhoea or other diagnoses (*p* = .000, Table 3), delaying entry to care by an average of 6.1 days (SD = 5.8). Children with diarrhoea were significantly more likely to enter care directly at the ED (*p* = .004). However, there were no significant differences by type of illness in the likelihood of ambulance transfer among those who were referred. Children with pneumonia had significantly higher acuity on arrival compared to children with diarrhoea and other diagnoses (*p* = .000). Children with diarrhoea tended to be less likely to be admitted to an ICU or die in hospital care, though these differences by illness type were not statistically significant.

**Table 3:** Care-seeking behaviours and outcomes among children with pneumonia (vs. other illnesses)

	Pneumonia (*N* = 132)	Diarrhoea (*N* = 53)	Other illnesses (*N* = 372)	*p*-value
Mean time from recognition of illness to seeking care outside the home (days) (SD)	6.1 days (5.8)	4.1 days (4.4)	3.2 days (4.9)	*p* = .000
Days delayed care seeking				*p* = .000
Less than 24 h	3 (2.3%)	0 (0.0%)	83 (22.3%)	
24–48 h	19 (14.4%)	14 (26.4%)	113 (30.4%)	
48–72 h	13 (9.9%)	12 (22.7%)	40 (10.7%)	
3–7 days	61 (46.2%)	20 (37.7%)	100 (26.9%)	
More than one week	36 (27.3%)	7 (13.2%)	36 (9.7%)	
Referred to VNCH from any lower-level facility	55 (41.7%)	9 (17.0%)	147 (39.6%)	*p* = .004
Transferred in ambulance if referred (N = 211)	38 (69.1%)	5 (55.6%)	79 (53.7%)	*p* = .143
Acuity on arrival				p = .000
ESI 1	17 (12.9%)	0 (0.0%)	42 (11.3%)	
ESI 2	59 (44.7%)	14 (26.4%)	91 (24.5%)	
ESI 3	56 (42.3%)	39 (73.6%)	239 (64.3%)	
Admitted to an ICU	12 (9.1%)	0 (0.0%)	37 (10.0%)	*p* = .057
Died in hospital	3 (2.3%)	0 (0.0%)	12 (3.2%)	*p* = .376

### Regression analysis

Children who were referred from another facility were significantly more likely to have higher acuity on arrival, compared to children who entered care at the VNCH directly (Table 4). Children referred from provincial hospitals had the highest odds of increased acuity, though those referred from lower-level facilities had similarly high odds for increased acuity on arrival (OR = 3.66, 95% CI 2.39–5.63; OR = 3.24, 95% CI 1.78–5.88, respectively). Children under age six, who are exempt from user fees, had significantly greater odds for more severe acuity on arrival (OR = 2.50, 95% CI 1.26–4.94). Pneumonia patients had significantly greater odds of increased acuity when examining risk of high versus moderate acuity (OR = 2.36, 95% CI 1.51–3.68), though pneumonia did not significantly predict risk for increased acuity for moderate versus highest acuity. Poverty significantly predicted increased acuity. Compared to the wealthiest children, the poorest had 4.98 times the odds of increased acuity (95% CI 1.82–13.61). Living in a rural area was marginally predictive of higher acuity, although children who are exempt from user fees and live in a rural area had significantly lower odds of increased acuity compared to other children (OR = 0.18, 95% CI 0.04–0.51).

**Table 4:** Adjusted odds of increasing acuity on arrival (*N* = 557)

		OR	95% CI
*Moderate to high acuity (ESI 3 to ESI 2)*	Child referred from provincial-level facility (vs. *no referral*)	3.66***	(2.39–5.63)
	Child referred from other type of facility (vs. *no referral*)	3.24***	(1.78–5.88)
	Days delayed care seeking outside the home	0.99	(0.96–1.03)
	Child is five years or younger (exempt from user fees)	2.50**	(1.26–4.94)
	Child is female	0.79	(0.54–1.15)
	Child has pneumonia (vs. *other diagnoses*)	2.36***	(1.51–3.68)
	Poorest wealth quintile (vs. *richest*)	4.98**	(1.82–13.61)
	Poorer wealth quintile (vs. *richest*)	1.29	(0.63–2.64)
	Middle wealth quintile (vs. *richest*)	1.14	(0.58–2.21)
	Richer wealth quintile (vs. *richest*)	1.87^+^	(0.97–3.61)
	Missing wealth quintile (vs. *richest*)	1.83	(0.82–4.08)
	Resides in a rural area	1.52^+^	(0.96–2.30)
	Mother attended secondary school (vs. *primary*)	0.78	(0.42–1.47)
	Mother attended post-secondary school (vs. *primary*)	0.99	(0.49–2.04)
	Child is exempt from user fees and from a rural area	0.18**	(0.04–0.51)
	Constant	0.11	
*High to highest acuity (ESI 2 to ESI 1)*	Child referred from provincial-level facility (vs. *no referral*)	3.66***	(2.39–5.63)
	Child referred from other type of facility (vs. *no referral*)	3.24***	(1.78–5.88)
	Days delayed care seeking outside the home	0.99	(0.96–1.03)
	Child is five years or younger (exempt from user fees)	2.50**	(1.26–4.94)
	Child is female	0.79	(0.54–1.15)
	Child has pneumonia (vs. *other diagnoses*)	1.14	(0.60–2.14)
	Poorest wealth quintile (vs. *richest*)	4.98**	(1.82–13.61)
	Poorer wealth quintile (vs. *richest*)	1.29	(0.63–2.64)
	Middle wealth quintile (vs. *richest*)	1.14	(0.58–2.21)
	Richer wealth quintile (vs. *richest*)	1.87^+^	(0.97–3.61)
	Missing wealth quintile (vs. *richest*)	1.83	(0.82–4.08)
	Resides in a rural area	1.52^+^	(0.96–2.30)
	Mother attended secondary school (vs. *primary*)	0.78	(0.42–1.47)
	Mother attended post-secondary school (vs. *primary*)	0.99	(0.49–2.04)
	Child is exempt from user fees and from a rural area	0.18**	(0.04–0.51)
	Constant	0.02	

***p < .001, **p < .01, *p < .05, ^+^ *p* < .10

## Discussion

This study describes referral and transfer patterns, predictors of acuity on arrival, and outcomes of children accessing a paediatric emergency department at a national referral hospital in a lower-income country, Vietnam. We found referrals from lower-level facilities were associated with higher acuity on arrival, greater likelihood of ICU admission, and greater risk of in-hospital mortality. Controlling for patient characteristics and care-seeking behaviours, the poorest children had significantly higher odds of increased acuity on arrival. The paediatric ED had a high burden of pneumonia patients, who were significantly more likely to have delayed entry into care, have entered care at a lower level, and had significantly higher acuity on arrival than patients with other conditions. While we found evidence of a functioning hierarchical referral system, our findings suggest emergency triage, referral, and care systems would benefit from initiatives to improve quality of care at primary and secondary facilities as well as better linkages among facilities, including the private sector. Such improvements contribute towards improved child health equity by increasing access to quality, appropriate emergency care for poorer and rural children.

The majority of patients were less than two years of age, and younger children experienced significantly higher acuity on arrival. Given that younger children face significantly higher risks for morbidity and mortality globally [34], this is unsurprising. The hospital’s patient population is predominantly male, which is common at tertiary facilities in Vietnam, and is potentially reflective of preferential care-seeking behaviours for male children [30, 39]. About half of children were from rural areas. That so many children from rural areas could access care at a national referral hospital is evidence that the referral system functions. However, it is problematic that less than 5% of patients attending the hospital are ethnic minorities, who comprise about 20% of Vietnam’s population [37]. Ethnic minorities in Vietnam are less likely to seek formal care outside the home [40]. This may be emblematic of a lack of benefit from equity schemes such as the user fee exemption, geographic isolation or systemic limitations in transport infrastructure, or additional barriers to care, such as difficulties navigating the health system [12]. Given that ethnic minorities have access to the same user fee exemption as Kinh children, targeted programs to identify and address the specific barriers to care faced by ethnic minority children may increase their access to higher-level care and existing health equity programs, reducing health disparities.

Referred patients were primarily referred by provincial hospitals, the next-highest level facility within the public health system. A low proportion of patients were referred by secondary public facilities, and none were referred by primary health centres. This suggests providers generally referred to sequentially higher facilities, only bypassing next-level facilities in rare cases. We found only a small number of referrals from private sector facilities to the highest-level public hospitals. Given that children across the wealth spectrum in Vietnam frequently utilize private-sector providers for treatment for fever, cough, and other illnesses [37], it may be that private providers informally refer patients to tertiary public facilities. However, without a formal referral, information about the patient may be lost in the transfer, potentially impacting quality of care. It may also be that private providers refer patients to primary or secondary public facilities, or to district or provincial hospitals. Alternatively, parents may be more likely to use public-sector care for higher acuity illnesses.

We identify several effects of inadequate referral systems. In an adjusted model, delays to entering care at any facility were not predictive of higher acuity on arrival, though being referred from a community, district, or provincial facility was significantly associated with higher acuity. It is important to note that it is the sickest children from community-level and district-level facilities who are referred to provincial and national hospitals. However, that referrals were significantly associated with higher acuity yet delays in entry to care were not may also signal issues of quality at lower-level facilities. This finding may also reflect problems with transport or referrals to national hospitals. Children referred from a lower-level facility were more likely to be admitted to an ICU and die in hospital care than those who entered care at the ED. Although children who were referred represent the most severely ill patients at lower-level facilities, inappropriate triage, ineffective stabilization, or poor recognition of severe illness and/or pneumonia at these facilities may be an important contributor to poor outcomes once these children reach district or provincial hospitals. Inadequate care during transfers may also contribute towards poorer outcomes among children who were referred to the ED from another facility. Among children who received ambulance transfers, staffing varied considerably. Prompt transfers with appropriate staffing and equipment can mitigate the impacts of long distances between facilities, and reduce barriers for families unable to secure transportation to tertiary facilities when referred [2, 41]. Overall, further investigation is necessary to identify how triage, recognition of illness, and quality of care at mid-level facilities in the Vietnamese context may contribute to delays within the health system.

We found that the poorest children had significantly higher acuity even after controlling for delay of entry to care, diagnosis, and other relevant characteristics. This suggests they may face additional barriers after entering care that contribute to further delays in reaching emergency care at national-level hospitals. However, among children who entered care at lower-level facilities, the poorest and ethnic minority children had a higher likelihood of ambulance transfer, mitigating transportation-related barriers. This may signify that these children were more likely to experience delays or other barriers to receiving care after arriving at community, district, or provincial facilities; or, alternately, that these children had higher acuity on arrival at these facilities, as we do not have data on acuity at arrival for referring facilities. The finding that the poorest children were significantly more likely to experience increased acuity on arrival may indicate that existing policies to address the needs of these children in accessing tertiary paediatric emergency care are insufficient. Notably, ethnic minority children are significantly less likely than Kinh children to access formal care in Vietnam, regardless of their household income level [40, 42]. Although children exempt from the user fee have significantly higher acuity on arrival, we assume this is the effect of younger age rather than the user fee itself because younger age was significantly correlated with higher acuity in our dataset. Encouragingly, children from rural areas who are exempt from user fees had significantly lower acuity, which is evidence that this policy improves access to advanced care for children from outlying areas who might otherwise experience geographic barriers to care. However, that being from a rural area tends to be correlated with higher acuity suggests distance may still present a significant barrier to care, consistent with other studies in Vietnam [41, 43]. Therefore, additional programs and/or subsidies may be necessary to improve equity by increasing access to advanced paediatric emergency care for children in rural or mountainous areas who live far from provincial or national hospitals.

The findings regarding pneumonia are of particular interest. Children with pneumonia were significantly more likely to have delayed entry to care, likely due to poor parental recognition of illness [44, 45]. Examining predictors of high versus moderate acuity, children with pneumonia had significantly greater risk for higher acuity than patients with other illnesses, controlling for other factors, though this risk was no longer significant when examining predictors of highest versus high acuity on arrival. Among children not in imminent danger of death, this suggests delay of entry to care does contribute to higher acuity for pneumonia in particular. However, children with pneumonia did not have higher odds of admission to an ICU or in-hospital mortality. Once these children are stabilized and appropriately treated, the need for intensive care decreases. Improving quality of care at primary and secondary facilities could reduce the burden of pneumonia patients at tertiary hospitals, and reduce time to appropriate care for children with pneumonia. Given that poorer children are significantly more likely to enter care at lower levels, such interventions may particularly benefit these children.

Our findings hold several implications for the improvement of paediatric emergency care in Vietnam and elsewhere. First, the quality of care at community, district, and even provincial facilities should be improved. Patients will benefit from better care at facilities more easily accessible to them, while health resources can be more appropriately distributed across facilities. From an equity perspective, such improvements may especially benefit poorer patients, minorities, and those in rural areas [40, 46]. Alternately, reducing bypassing behaviour will also improve health equity. Richer children can more easily afford the fee that allows them to bypass primary and second-level facilities to enter care at referral hospitals. Encouraging greater use of primary and secondary facilities will help reserve advanced health resources for the children who need them most. Currently, no well-developed triage system exists in community or district-level facilities in Vietnam, which would aid in identifying most at risk children. Provider training in triage and critical care at all levels is necessary to improve quality of paediatric emergency care, including consistent implementation of international guidelines, such as the ETAT guidelines and guidelines for pneumonia care [16]. Efforts to improve stabilization of critically ill children prior to transport may lead to lower acuity on arrival at higher-level facilities. Strengthening referral systems can improve outcomes regardless of where a child enters care, and regardless of his or her family’s ability to pay. Finally, improving linkages between public and private sectors may improve outcomes for children who enter care with private providers.

Taken together, efforts to improve triage, referrals, and provider skills can significantly improve the quality of paediatric emergency care in low- and middle-income countries, and increase access to advanced care in these settings. In tertiary hospitals in Kenya and Malawi, strategies such as introducing a triage system, increased supervision for emergency care, uptake of ETAT guidelines, and treating and stabilizing patients prior to transfers to wards at a paediatric hospital resulted in a significant decrease in early and overall mortality [47]. Improved quality of critical care at tertiary referral facilities will also benefit emergently ill children who are admitted to ICUs [5]. These and other interventions [48] highlight the importance of combining provider training with organizational changes to sustainably improve quality of emergency and triage care. However, improvements to the health system alone will not reduce health disparities [49]. Given that current subsidy schemes in Vietnam have improved utilization of care among the poor, it is important to address barriers to access and determinants of delay beyond costs of care [31, 32].

Our study has several limitations. We did not capture children who did not access care outside the home, or those who did not reach tertiary care. Further research on children who do not attend formal care and those who complete care at community or district facilities or private clinics is necessary to capture a system-wide view of paediatric emergency care. We did not include children dying or in imminent danger of death in our sampling frame for ethical reasons, who may systematically differ from children in the sample. Because we do not have medical or socio-demographic information on these children, we cannot assess the magnitude of bias introduced by this exclusion. About 16% of children are missing wealth information. We were unable to measure distance to care, which is known to be a key determinant of access in Vietnam and globally [43]. Future studies should actively consider the role of distance to primary, secondary, and tertiary facilities. Finally, we were unable to obtain detailed information on medical care received at referring hospitals and clinics, which would inform concerns about poor quality of care at referring facilities. We also lack data on referrals to facilities attended prior to the facility referring patients to VNCH. Therefore, future research should sample across health systems to identify factors related to drop out and care at lower levels, and to characterize the effects of referrals throughout the spectrum of available health services.

## Conclusions

Advanced paediatric emergency care is understudied in many low- and middle-income countries, even in increasingly complex health systems. Our data provide insights into referral patterns, outcomes, and equity for children accessing highest-level paediatric emergency care in a lower-income country, Vietnam, including for pneumonia. We find that poorer children are more likely to experience higher acuity on arrival at national-level hospitals, which may signal issues of quality at referring facilities. Addressing health equity by making improvements to the referral system and care at lower levels might benefit the poorest children in particular, who are significantly more likely to enter care at primary or secondary facilities.

Vietnam has a functioning paediatric referral system, though systems-level improvements in paediatric care at all levels would help ensure timely access to quality emergency care for all children, regardless of where they first access care. Quality, accessible paediatric emergency care constitutes an important part of health systems. Better triage, stabilization, and referral guidelines, and transport systems linking all levels of care should result in improved quality of paediatric emergency care. Such improvements to paediatric emergency and critical care throughout Vietnam’s health system will contribute to further reductions in child morbidity and mortality, and improved child health equity at a population level.

## Abbreviations

ED: Emergency Department
ESI: Emergency Severity Index
ICU: Intensive Care Unit
VNCH: Vietnam National Children’s Hospital
